# Supplementary material for: Content-rich biological network constructed by mining PubMed abstracts
Source: BMC Bioinformatics. 2004 Oct 8;5:147. doi: 10.1186/1471-2105-5-147 (PMC528731; doi:10.1186/1471-2105-5-147)
Supplement: Additional File 2 — The original results of the above study (non-essential files are deleted to keep the file size under the limit set by BMC bioinformatics). [file 1471-2105-5-147-S2.bz2 › chilibotAdditionalFile2/dip05/369ID9045692E6043/html/FGFR3_FGF5.html]

 


 **FGFR3** and **FGF5** 
  
Found 5 abstracts in PubMed, retrieved 05.  
 

 What does Google say? 
 PDF only 
| .edu only 

---

- J Histochem Cytochem, 2000   **Gene expression patterns of the fibroblast growth factors and their receptors during myogenesis of rat satellite cells.** .
  Satellite cells are the myogenic precursors in postnatal muscle and are situated beneath the myofiber basement membrane.
  We previously showed that fibroblast growth factor 2 FGF2, basic FGF stimulates a greater number of satellite cells to enter the cell cycle but does not modify the overall schedule of a short proliferative phase and a rapid transition to the differentiated state as the satellite cells undergo myogenesis in isolated myofibers.
  In this study we investigated whether other members of the FGF family can maintain the proliferative state of the satellite cells in rat myofiber cultures.
  We show that FGF1, FGF4, and FGF6 as well as hepatocyte growth factor, HGF enhance satellite cell proliferation to a similar degree as that seen with FGF2.
  . . . **FGF5** and FGF7 are ineffective.
  None of the growth factors prolongs the proliferative phase or delays the transition of the satellite cells to the differentiating, myogenin state.
  However, FGF6 retards the rapid exit of the cells from the myogenin state that routinely occurs in myofiber cultures.
  To determine which of the above growth factors might be involved in regulating satellite cells in vivo, we examined their mRNA expression patterns in cultured rat myofibers using RT PCR.
  The expression of all growth factors, excluding FGF4, was confirmed.
  Only FGF6 was expressed at a higher level in the isolated myofibers and not in the connective tissue cells surrounding the myofibers or in satellite cells dissociated away from the muscle.
  By Western blot analysis, we also demonstrated the presence of FGF6 protein in the skeletal musle tissue.
  Our studies therefore suggest that the myofibers serve as the main source for the muscle FGF6 in vivo.
  We also used RT PCR to analyze the expression patterns of the four tyrosine kinase FGF receptors FGFR1 FGFR4 and of the HGF receptor c met in the myofiber cultures.
  Depending on the time in culture, expression of all receptors was detected, with FGFR2 and **FGFR3** expressed only at a low level.
  Only FGFR4 was expressed at a higher level in the myofibers but not the connective tissue cell cultures.
  FGFR4 was also expressed at a higher level in satellite cells compared to the nonmyogenic cells when the two cell populations were released from the muscle tissue and fractionated by Percoll density centrifugation.
  The unique localization patterns of FGF6 and FGFR4 may reflect specific roles for these members of the FGF signaling complex during myogenesis in adult skeletal muscle.

  - Glia, 2000   **Fibroblast growth factors 5 and 9 distinctly regulate expression and function of the gap junction protein connexin43 in cultured astroglial cells from different brain regions.** .
    Astroglial cells contribute to neuronal maintenance and function in the normal and diseased brain.
    Gap junctions formed predominantly by connexin43 cx43 provide important pathways to coordinate astroglial responses.
    We have previously shown that fibroblast growth factor FGF 2, which occurs ubiquitously in the CNS, downregulates gap junction communication in cortical and striatal, but not in mesencephalic astroglial cells in vitro Reuss et al.
    Glia 22 19 3 998 .
    Other members of the FGF family expressed in the CNS include FGF 5 **[FGF5]** and FGF 9.
    We show that both FGF 5 **[FGF5]** and FGF 9, like FGF 2, downregulate astroglial gap junctions and functional coupling.
    However, their effects are strikingly different from different brain regions, with regard to astroglial cells.
    FGF 5 **[FGF5]** specifically affects mesencephalic astroglial cells without changing coupling of cortical and striatal astroglia, while FGF 9 reduces gap junctional coupling in astroglia from all three brain regions.
    Both cx43 mRNA and protein levels as well as functional coupling assessed by dye spreading are affected.
    To clarify whether brain region specific effects of FGFs on astroglial coupling are due to differential expression of FGF receptors FGFR , we monitored expression of the four known FGFR mRNAs in astroglial cultures by RT PCR.
    Irrespective of their regional origin, astroglial cells express mRNAs for FGFR 2 and FGFR 3 **[FGFR3]**.
    In summary, our results provide evidence for an important role of FGF , and 9 in a distinct, CNS region specific regulation mechanism of astroglial gap junction communication.
    The molecular basis underlying the regionally distinct responsiveness of astrocytes to different FGFs may be sought beyond distinct FGFR expression.

    - Kidney Int, 1999   **Expression and localization of fibroblast growth factors and fibroblast growth factor receptors in the developing rat kidney.** .
      Expression and localization of fibroblast growth factors and fibroblast growth factor receptors in the developing rat kidney.
      BACKGROUND The permanent kidney, or metanephros, develops through a complex series of reciprocal inductive events and involves branching morphogenesis, tubulogenesis, angiogenesis, and tissue remodeling.
      Fibroblast growth factors FGFs are a family of growth and differentiation factors that have been implicated in metanephric development.
      FGFs exert their actions through tyrosine kinase receptors, FGFRs, which are encoded by four FGFR genes FGFR1 through FGFR4 .
      METHODS Reverse transcriptase polymerase chain reaction was used to detect the expression of FGFs and FGFRs in rat metanephroi from embryonic day E 14 to E21.
      Nonradioactive in situ hybridization was used to localize FGF1 mRNA in E20 rat metanephroi, and immunohistochemistry was used to localize FGFRs in E15 and E20 rat metanephroi.
      RESULTS We detected the expression of mRNAs for FGF1 through **FGF5**, FGF7 through FGF10, and FGFR1 through FGFR4 IIIb and IIIc splice variants in rat metanephroi from E14 to E21.
      By in situ hybridization, FGF1 mRNA was detected in the nephrogenic zone, ureteric epithelium, and developing nephron elements.
      FGFR proteins were localized in a distinct pattern that altered with maturation.
      FGFR1 was widely distributed in developing metanephric epithelia and mesenchyme, but not in developing interstitium.
      FGFR2 was also widely distributed in nephron epithelia, particularly in proximal convoluted tubules, but was not detected in metanephric mesenchyme, mesenchymal condensates, or developing interstitium.
      **FGFR3** was localized to mesenchymal condensates, nephron elements, and medullary interstitium but not proximal convoluted tubules.
      FGFR4 was localized mostly to maturing nephron structures and was not detected in nephrogenic mesenchyme, mesenchymal condensates, or developing interstitium.
      CONCLUSIONS These results indicate that FGFs and FGFRs are expressed in the developing rat metanephros from at least E14.
      they likely play important roles in metanephric development and maturation.

      - Dev Dyn, 1996   **Fibroblast growth factor signalling in the hair growth cycle expression of the fibroblast growth factor receptor and ligand genes in the murine hair follicle.** .
        Using RNA in situ hybridization analysis, we have characterized the expression domains of the four known members of the FGF receptor tyrosine kinase gene family in the murine hair follicle at various stages of the hair growth cycle.
        During anagen, we detected Fgfr1 RNA in the dermal papilla, Fgfr2 RNA in hair matrix cells near the dermal papilla, **Fgfr3** RNA in pre cuticle cells in the periphery of the hair bulb, and Fgfr4 RNA in cells in the periphery of the hair bulb and also in the inner and outer root sheath in the lower half of the follicle neck.
        No RNA expression of these genes was detected during late catagen or telogen.
        We have previously shown that **Fgf5** is expressed in the outer root sheath in the transient portion of the follicle Hébert et al.
        1994 Cell 78 1017 1025 .
        In the present study we have also assayed for the expression of six other members of the FGF ligand gene family, Fgf3, Fgf4, Fgf6, Fgf7, Fgf8, and Fgf9.
        Among these FGF genes, only Fgf7 was found to be expressed in the hair follicle.
        Fgf7 RNA is localized to the dermal papilla during anagen, but expression is down regulated by the late anagen VI stage.
        We have also demonstrated that addition of **FGF5** protein to the culture medium changes the behavior of dermal papilla cells in vitro, indicating that they are capable of responding to **FGF5**.
        Together with previously published data, these results provide a complete analysis of FGF ligand and FGF receptor tyrosine kinase gene expression in the hair follicle, and suggest that FGF signalling may have several functions in the hair growth cycle.

        - Int J Cancer, 1995   **Expression of FGF and FGF receptor genes in human breast cancer.** .
          The family of FGF growth factors is involved in several biological processes and might play an important role in tumorigenesis.
          We have studied the respective expression of 8 of the 9 characterized FGF genes, and of the 4 known FGF receptor genes, in a panel of 10 tumor cell lines and 103 breast tumor samples, using RT PCR and Northern blot analyses.
          FGF1 and FGF2 were expressed in almost all samples, while expression of **FGF5**, FGF6, FGF7, and FGF9 was more restricted.
          FGFR1, FGFR2 and FGFR4 were expressed at high levels in respectively 22%, 4% and 32% of tumors.
          **FGFR3** expression was not detected.
          The transcript encoding an FGFR1 isoform with 2 immunoglobulin like domains was the most prevalent.
